# Supplementary figures and images for: Antidiarrheal, analgesic,antidepressant, antimicrobial and hypoglycemic activities of methanolic extract from Sonneratia apetala fruit, with identification of bioactive compounds in n-hexane, chloroform, and ethyl acetate fractions
Source: PLoS One. 2025 May 5;20(5):e0321280. doi: 10.1371/journal.pone.0321280 (PMC12052150; doi:10.1371/journal.pone.0321280)

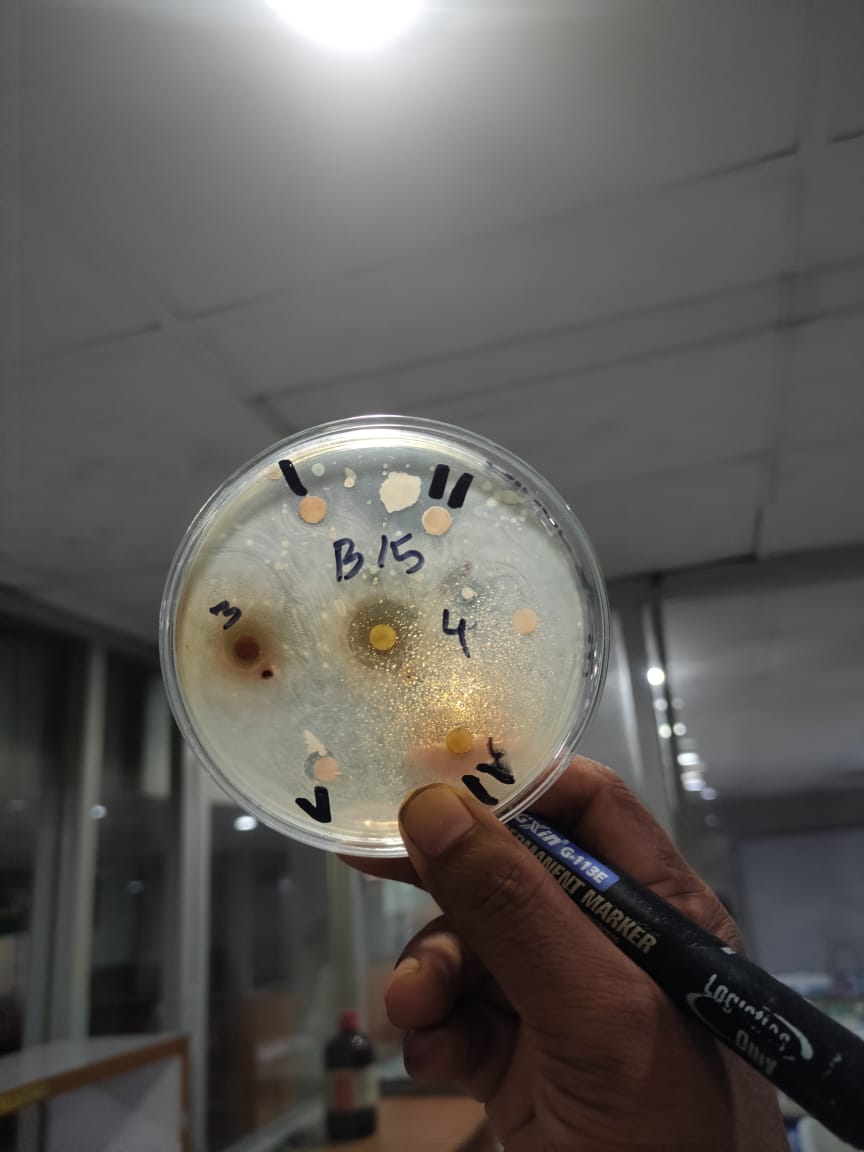

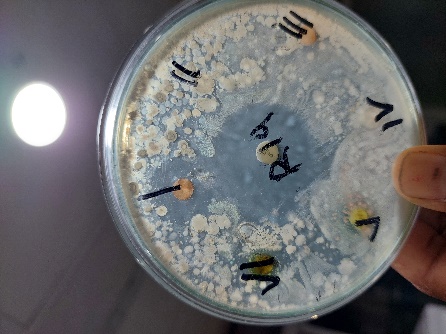

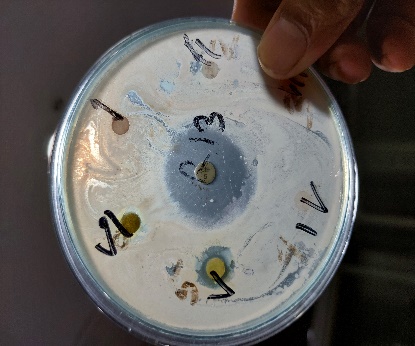

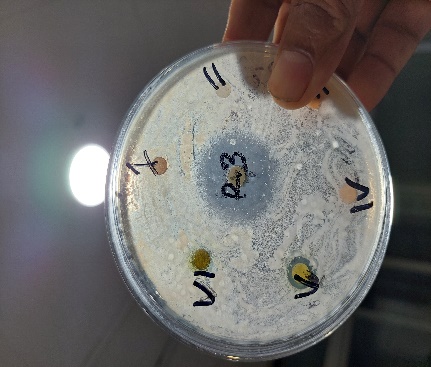


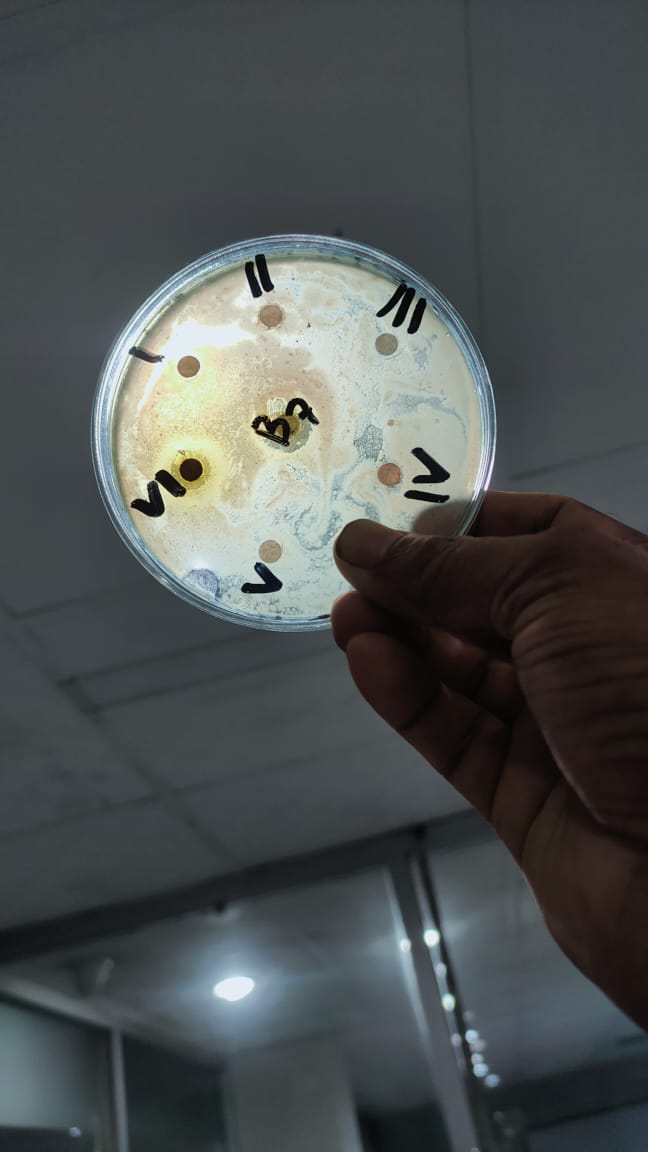

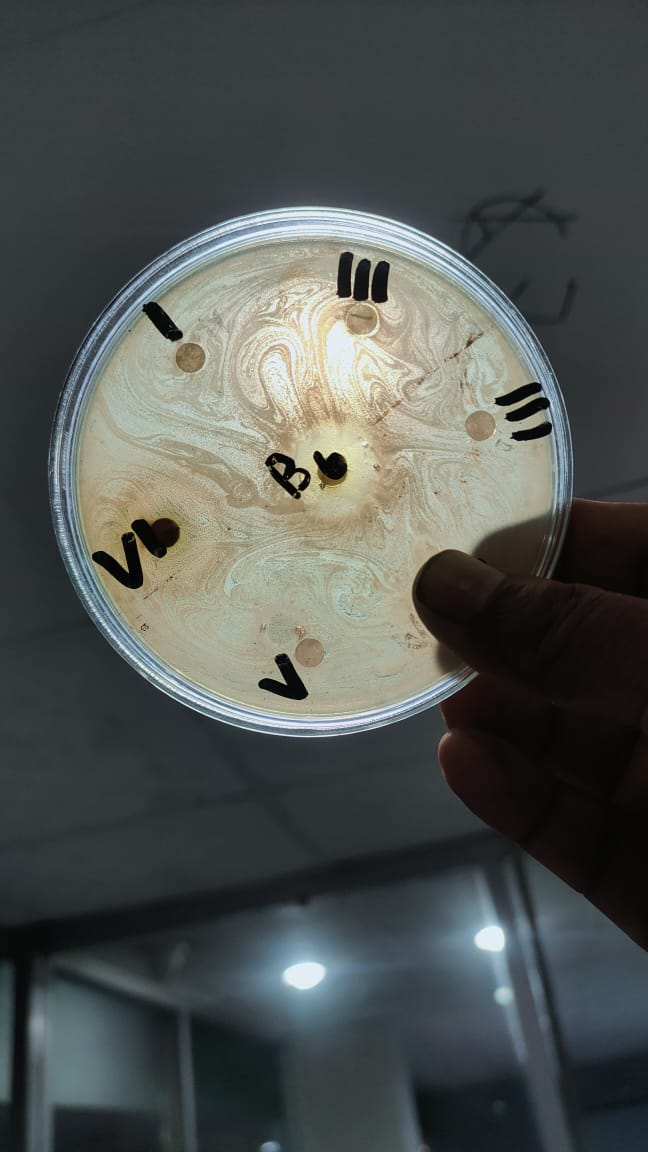

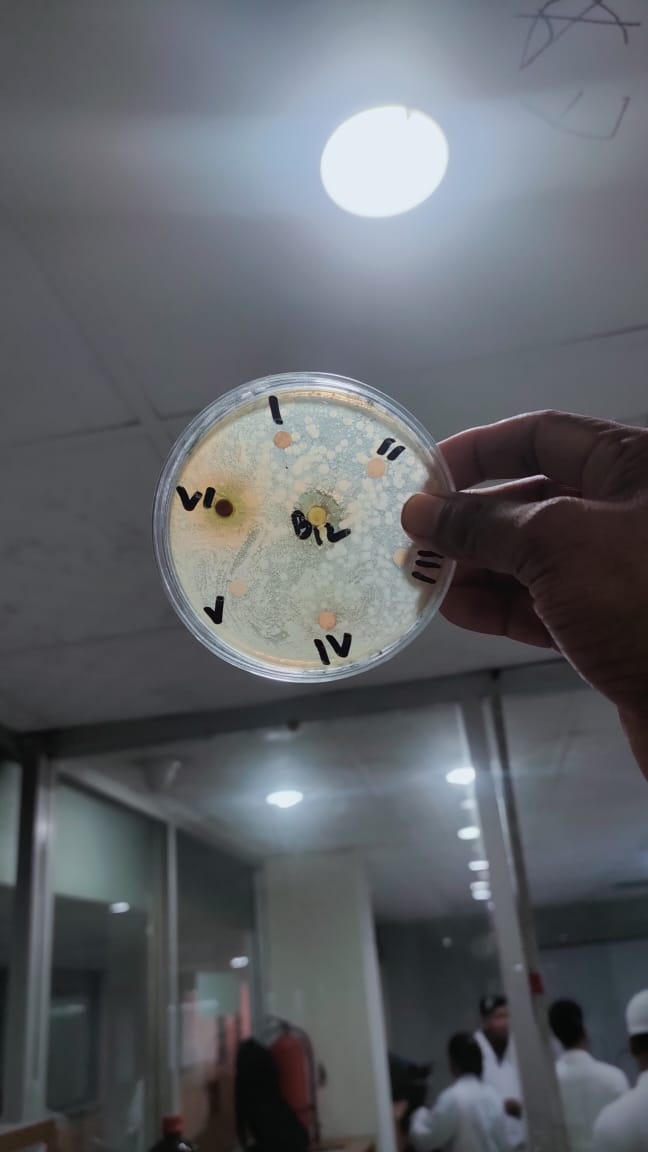

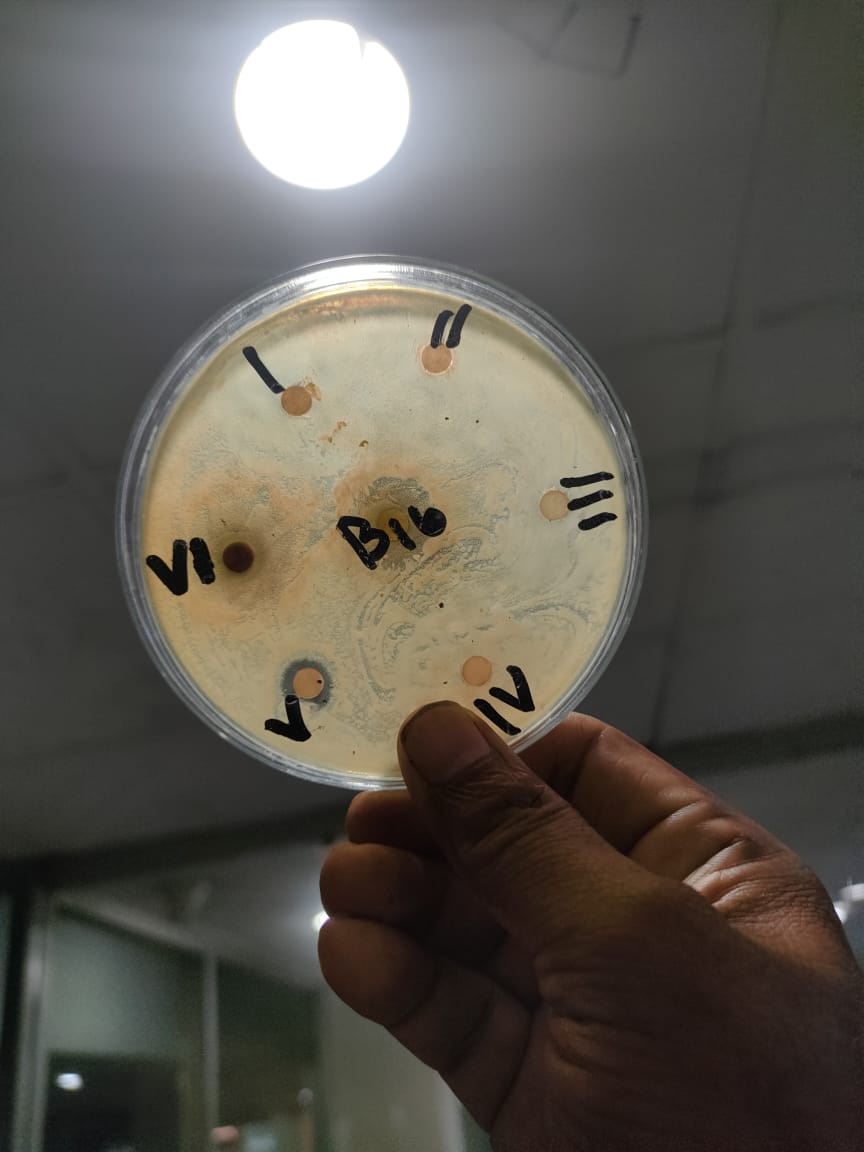


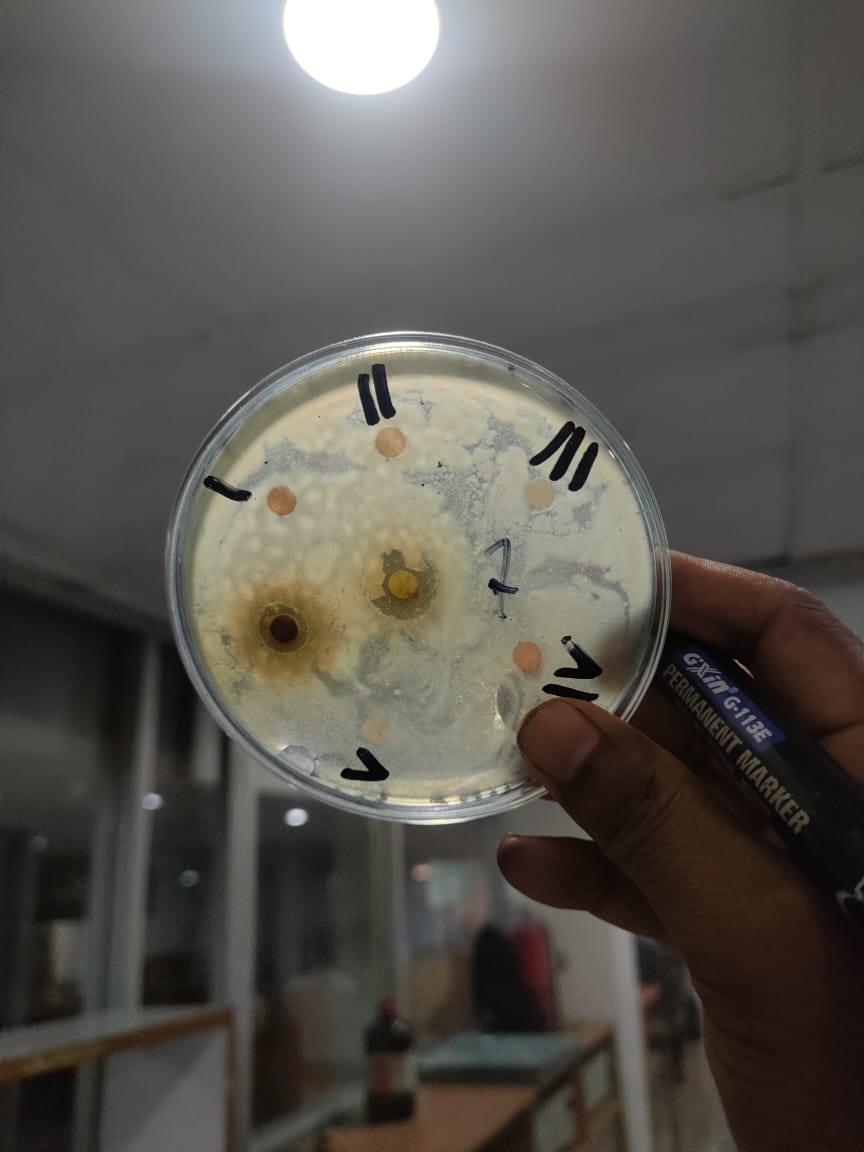

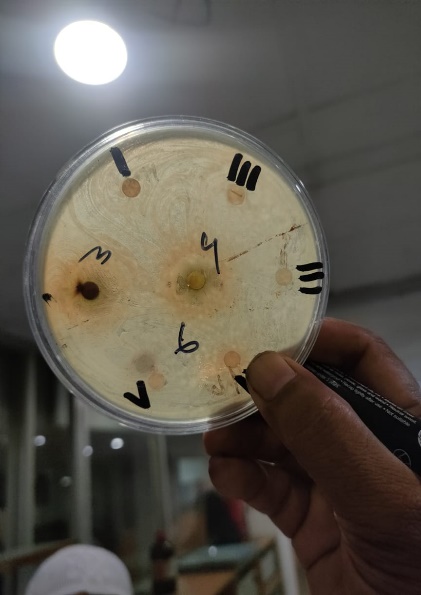


Fig. S1. Antimicrobial activity of methanolic extracts of the pericarp and seed of *S. apetala*

Supplement: Fig S1 — (DOCX) [file pone.0321280.s001.docx]
