# Supplementary material for: Antidiarrheal, analgesic,antidepressant, antimicrobial and hypoglycemic activities of methanolic extract from Sonneratia apetala fruit, with identification of bioactive compounds in n-hexane, chloroform, and ethyl acetate fractions
Source: PLoS One. 2025 May 5;20(5):e0321280. doi: 10.1371/journal.pone.0321280 (PMC12052150; doi:10.1371/journal.pone.0321280)
Supplement: Table S1 — (DOCX) [file pone.0321280.s002.docx]

Table S1. Evaluation of the central analgesic activity of a crude extract of *Sonneratia apetala* pericarp and seed

| group | Dose (mg/kg bw) | Immersion time counts (second) *MESP* | | | | Immersion time counts (second) *MESS* | | | |
| --- | --- | --- | --- | --- | --- | --- | --- | --- | --- |
|  |  | 0 min | 30 min | 60 min | 90 min | 0 min | 30 min | 60 min | 90 min |
| CTL | 0 | 2.06±0.35 | 2.28±0.23 | 2.27±0.209 | 2.85±0.27 | 2.06±0.35 | 2.28±0.23 | 2.27±0.209 | 2.85±0.27 |
| STD | 2 | 1.86±0.34 | 5.55±0.19 | 10.12±0.33 | 17.84±0.41 | 1.86±0.34 | 5.55±0.19 | 10.12±0.33 | 17.84±0.41 |
| MESF | 200 | 2.06±0.17 | 3.69±0.15 | 5.30±0.22 | 7.40±0.27 | 2.27±0.40 | 6.62±0.19 | 5.4±0.38 | 7.79±0.23 |
| MESF | 400 | 2.15±0.21 | 4.50±0.18 | 7.29±0.73 | 10.16±0.59 | 2.11±0.33 | 4.06±0.23 | 6.04±0.42 | 8.18±0.38 |
