# Supplementary material for: Antidiarrheal, analgesic,antidepressant, antimicrobial and hypoglycemic activities of methanolic extract from Sonneratia apetala fruit, with identification of bioactive compounds in n-hexane, chloroform, and ethyl acetate fractions
Source: PLoS One. 2025 May 5;20(5):e0321280. doi: 10.1371/journal.pone.0321280 (PMC12052150; doi:10.1371/journal.pone.0321280)
Supplement: Table S2 — (DOCX) [file pone.0321280.s003.docx]

Table S2. Effect of methanolic extract of *S. apetala* pericarp and seed on castor oil induced diarrhea in mice

| Time | Group | Avg. NO. of feces of *MESP* | % reduction of diarrhea of *MESP* | Avg. NO. of feces of *MESS* | % reduction of diarrhea of *MESS* |
| --- | --- | --- | --- | --- | --- |
| After 1 hour | CTL | 2.25 | - | 2.25 | - |
|  | STD | 0.0 | 100 | 0.0 | 100 |
|  | MESF (200) | 0.5 | 77.78 | 0.75 | 66.67 |
|  | MESF (400) | 0.75 | 66.67 | 0.25 | 88.89 |
| After 2 hours | CTL | 5.0 | - | 5.0 | - |
|  | STD | 0.25 | 95 | 0.25 | 95 |
|  | MESF (200) | 0.5 | 90 | 2.25 | 55 |
|  | MESF (400) | 1.0 | 80 | 1.50 | 70 |
| After 3 hours | CTL | 8 | - | 8.0 | - |
|  | STD | 0.17 | 97.87 | 0.17 | 97.87 |
|  | MESF (200) | 1.75 | 78.13 | 3.0 | 62.5 |
|  | MESF (400) | 1.75 | 78.13 | 3.75 | 53.125 |
| After 4 hours | CTL | 10.75 | - | 10.75 | - |
|  | STD | 2.0 | 81.39 | 2.0 | 81.39 |
|  | MESF (200) | 3.50 | 67.44 | 4.50 | 58.13 |
|  | MESF (400) | 3.0 | 72.09 | 5.75 | 46.51 |
