# Supplementary material for: Antidiarrheal, analgesic,antidepressant, antimicrobial and hypoglycemic activities of methanolic extract from Sonneratia apetala fruit, with identification of bioactive compounds in n-hexane, chloroform, and ethyl acetate fractions
Source: PLoS One. 2025 May 5;20(5):e0321280. doi: 10.1371/journal.pone.0321280 (PMC12052150; doi:10.1371/journal.pone.0321280)
Supplement: Table S3 — (DOCX) [file pone.0321280.s004.docx]

Table S3. Anti-depressant activity of *S. apetala* by Thiopental sodium induced sleeping time method for pericarp and seed

| Group |  | Anti-depressant activity of MESP | | | Anti-depressant activity of MESS | |
| --- | --- | --- | --- | --- | --- | --- |
|  | Dose (mg/kg bw) | | Avg. time of onset of sleep (min.) | Avg. of total sleeping time (min.) | Avg. time of onset of sleep (min.) | Avg. of total sleeping time (min.) |
| CTL | 0 | | 39.75 | 79.75 | 39.75 | 79.75 |
| STD | 2 | | 39.25 | 68.5 | 39.25 | 68.5 |
| MESF | 200 | | 63.25 | 34.5 | 56.25 | 36 |
| MESF | 400 | | 57.75 | 37 | 52 | 49.25 |
