# Supplementary material for: Antidiarrheal, analgesic,antidepressant, antimicrobial and hypoglycemic activities of methanolic extract from Sonneratia apetala fruit, with identification of bioactive compounds in n-hexane, chloroform, and ethyl acetate fractions
Source: PLoS One. 2025 May 5;20(5):e0321280. doi: 10.1371/journal.pone.0321280 (PMC12052150; doi:10.1371/journal.pone.0321280)
Supplement: Table S4 — (DOCX) [file pone.0321280.s005.docx]

Table S4. Plasma level of glucose (mmol/L) of mice at different times for the pericarp and seed of S. apetala

| Group | Dose (mg/kg bw) | Plasma level of glucose (mmol/L) of MESP | | | | Plasma level of glucose (mmol/L) of MESS | | | |
| --- | --- | --- | --- | --- | --- | --- | --- | --- | --- |
|  |  | 0 min | 60 min | 120 min | 180 min | 0 min | 60 min | 120 min | 180 min |
| CTL | 0 | 8.4 | 19.45 | 14.77 | 12.43 | 8.4 | 19.45 | 14.77 | 12.43 |
| STD | 2 | 8.57 | 13.92 | 8.65 | 4.3 | 8.57 | 13.92 | 8.65 | 4.3 |
| MESF | 200 | 7.75 | 11.43 | 8.5 | 7.35 | 8.28 | 10.7 | 8.7 | 7.25 |
| MESF | 400 | 8.95 | 16.43 | 14.08 | 10.28 | 8.0 | 11.5 | 8.58 | 7.68 |
