# Supplementary material for: Antidiarrheal, analgesic,antidepressant, antimicrobial and hypoglycemic activities of methanolic extract from Sonneratia apetala fruit, with identification of bioactive compounds in n-hexane, chloroform, and ethyl acetate fractions
Source: PLoS One. 2025 May 5;20(5):e0321280. doi: 10.1371/journal.pone.0321280 (PMC12052150; doi:10.1371/journal.pone.0321280)
Supplement: Attachment — Submitted filename: PONE-D-24-48754_removed (1).pdf [file pone.0321280.s007.pdf]

Bangla Desh

article

Quick Submit

Quick Submit

University of Karachi

Document Details

Submission ID

trn:oid:::1:3126387390

Submission Date

Jan 9, 2025, 11:58 AM GMT+5

Download Date

Jan 9, 2025, 12:00 PM GMT+5

File Name

PONE-D-24-48754_removed.pdf

File Size

1006.5 KB

19 Pages

7,702 Words

41,390 Characters

29% detected as AI

The percentage indicates the combined amount of likely AI-generated text as well as likely AI-generated text that was also likely AI-paraphrased.

Caution: Review required.

It is essential to understand the limitations of AI detection before making decisions about a student's work. We encourage you to learn more about Turnitin's AI detection capabilities before using the tool.

Detection Groups

1 AI-generated only 29%
Likely AI-generated text from a large-language model.

2 AI-generated text that was AI-paraphrased 0%
Likely AI-generated text that was likely revised using an AI-paraphrase tool or word spinner.

Disclaimer
Our AI writing assessment is designed to help educators identify text that might be prepared by a generative AI tool. Our AI writing assessment may not always be accurate (it may misidentify writing that is likely AI generated as AI generated and AI paraphrased or likely AI generated and AI paraphrased writing as only AI generated) so it should not be used as the sole basis for adverse actions against a student. It takes further scrutiny and human judgment in conjunction with an organization's application of its specific academic policies to determine whether any academic misconduct has occurred.

Frequently Asked Questions

How should I interpret Turnitin's AI writing percentage and false positives?
The percentage shown in the AI writing report is the amount of qualifying text within the submission that Turnitin's AI writing detection model determines was either likely AI-generated text from a large-language model or likely AI-generated text that was likely revised using an AI-paraphrase tool or word spinner.

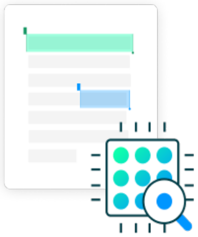

False positives (incorrectly flagging human-written text as AI-generated) are a possibility in AI models.

AI detection scores under 20%, which we do not surface in new reports, have a higher likelihood of false positives. To reduce the likelihood of misinterpretation, no score or highlights are attributed and are indicated with an asterisk in the report (*%).

The AI writing percentage should not be the sole basis to determine whether misconduct has occurred. The reviewer/instructor should use the percentage as a means to start a formative conversation with their student and/or use it to examine the submitted assignment in accordance with their school's policies.

What does 'qualifying text' mean?
Our model only processes qualifying text in the form of long-form writing. Long-form writing means individual sentences contained in paragraphs that make up a longer piece of written work, such as an essay, a dissertation, or an article, etc. Qualifying text that has been determined to be likely AI-generated will be highlighted in cyan in the submission, and likely AI-generated and then likely AI-paraphrased will be highlighted purple.

Non-qualifying text, such as bullet points, annotated bibliographies, etc., will not be processed and can create disparity between the submission highlights and the percentage shown.

Manuscript

Click here to access/download;Manuscript;Manuscripr_kaora.docx

Antidiarrheal, Antidepressant, Hypoglycemic, Analgesic, and Antimicrobial Activities of Methanolic Extract from *Sonneratia apetala* Fruit, with Identification of Bioactive Compounds in n-Hexane, Chloroform, and Ethyl Acetate Fractions

Fatema Akhter[1], Md. Ripaj Uddin[2*], Muhammad Abdullah Al Mansur[2], Mohammad Saydur Rahman[3], Md. Ahedul Akbor[2], Nahida Akter[2], Abubakr M. idris[4], Golam Mostafa[5], AHM Shofiul Islam Molla Jamal[2], Sarker Kamruzzaman[2]

[1]*Department of Pharmacy, Bangladesh University, Dhaka, Bangladesh; fatemaaktherjenny@gmail.com (FA)*

[2] *Institute of National Analytical Research and Service (INARS), Bangladesh Council of Scientific and Industrial Research (BCSIR), Dhanmondi, Dhaka 1205, Bangladesh; md.ripajuddin@gmail.com (MRU); nayeembcsir@gmail.com (MAAM); nahida.nipaimsf@gmail.com (NA); dir-inars@bcsir.gov.bd (SK).*

[3]*Department of Pharmacy, Jagannath University, Dhaka, Bangladesh; saydur_pr@yahoo.com (MSR)*

[4]*Department of Chemistry, King Khalid University, College of Science, P.O. Box 9004, Abha 62529, Saudi Arabia;dramidris@gmail.com(ABME)*

[5]*Institute of Mining, Mineralogy and Metallurgy, Bangladesh Council of Scientific and Industrial Research, Joypurhat-5900, Bangladesh*

[*]Corresponding author: E-mail address: md.ripajuddin@gmail.com (M.R.U); *nayeembcsir@gmail.com (MAAM)*

Abstract

The rapidly growing mangrove fruit *Sonneratia apetala*, native to the deltaic region of Bangladesh, holds promise in traditional medicine due to its bioactivity and antimicrobial properties. Sample collections from Nijum Dwip, Hatiya, and Noakhali in Bangladesh were divided into pericarps and seeds, subsequently fractionated with methanol, n-hexane, ethyl acetate, and chloroform. Bioactivity assays involved Swiss albino mice, acquired from ICDDR, B, in compliance with FELASA standards. Standard agents such as diclofenac sodium, loperamide, diazepam, and glibenclamide were used to evaluate antidiarrheal, antidepressant, hypoglycaemic, and analgesic effects, while ciprofloxacin served as a reference for antibacterial and antifungal testing. Methanolic extracts (ME) of the seed and pericarp exhibited notable central and peripheral analgesic effects at 200 and 400 mg/kg dosages. The ME of seeds demonstrated the strongest antidiarrheal efficacy at 400 mg/kg after 1 hour, and the pericarp at 200 mg/kg after 2 hours. The ME also showed significant antidiabetic potential in 99% seed and pericarp extracts. GC-MS analysis revealed seven bioactive compounds in the n-hexane, ethyl acetate, and chloroform fractions, including N-Ethyl-2-methylbenzenesulfonamide, 3,6-Pyridazinedione, 1,2-dihydro-1-(4-nitrophenyl), N-Acetyl-alpha-aminooxybutyric acid (methyl), 2H-Phenanthro[2,1-b] azepin-2-one (1,3,4,5,5a), and Undecane. These compounds have established

anticancer and antimicrobial properties. Both pericarp and seed extracts displayed strong antifungal activity against *Saccharomyces cerevisiae*, *Candida albicans*, and *Aspergillus niger*, while moderate antibacterial effects were noted against gram-negative strains like *Salmonella Typhi* and *Pseudomonas aeruginosa*, as well as gram-positive bacteria such as *Staphylococcus aureus*. These findings underscore *S. apetala*'s potential as a valuable bioactive source for traditional medicinal applications.

***Keywords*:** Antidiarrheal, Antidepressant, Hypoglycemic, Analgesic and Antimicrobial

1. Introduction

Sonneratia apetala Buch. Ham. is a rapidly spreading mangrove species characterized by its quadrangular branches, commonly found along the coastlines of Bangladesh, Australia, Malaysia, India and other countries [1]. These plants play a crucial role in natural ecosystems and thrive in newly accreted soil with moderate to high salinity levels [2]. The fruits of *S. apetala* have a durable leathery calyx and measure 1.5 to 2 cm in diameter, containing a rich array of vitamins, especially Vitamin C, minerals, and various other nutrients. Each fruit contains both pericarp and seeds (see Figure 1). The pericarp is extremely thin and holds numerous small seeds that combine to form a single large seed. The seeds are yellowish and arranged tightly in 6 to 8 locules, primarily shaped like a U or V [3]. Polyphenols, anthocyanins, flavonoids, and Vitamin C have been identified in the fruit extract of *S. apetala*. The high concentration of flavonoids, polyphenols, and anthocyanins, which are functional components, contributes to the fruit extract's remarkable antibacterial properties [4]. Coastal communities utilise *Sonneratia apetala* fruit juice as a tonic to alleviate conditions such as diarrhoea [5]. *S. apetala* also possesses potent antimicrobial, antioxidant, and cytotoxic properties (Islam et al. 2013). The aqueous extract of the green fruit of the Keora (*S. apetala*) efficiently catalyzes the Knoevenagel condensation reaction involving aromatic aldehydes and malononitrile, resulting in a higher yield of arylidene-malononitrile synthesis. Because this fruit's aqueous extract is entirely eco-friendly and biodegradable, it holds significant potential as a candidate for green chemistry applications as a catalyst.

Diarrhoea remains a significant public health challenge, especially in developing regions where access to clean drinking water and adequate sanitation is often restricted. Numerous studies have demonstrated the potential of plant-based treatments to address diarrhoea, and *S. apetala* is one such plant with promising results. The ethanolic extract of *S. apetala* fruit has been shown to significantly reduce diarrhoeal episodes in animal models, primarily through its ability to modulate gut motility and decrease intestinal secretions [6]. The presence of tannins and flavonoids, which are known to have astringent properties, is thought to contribute to this antidiarrheal effect by reducing intestinal fluid

loss and improving gut health [7]. Depression is a prevalent and disabling mental health disorder impacting millions globally. Although pharmaceutical treatments are available, their associated side effects have driven a growing interest in alternative therapies. The ethanolic extract of *S. apetala* fruit has shown antidepressant-like activity in animal models, possibly by enhancing the levels of neurotransmitters such as serotonin and dopamine [1]. The bioactive compounds in *S. apetala* are thought to act on the central nervous system, thereby influencing mood regulation. This suggests that the fruit extract could be a potential candidate for developing natural antidepressant drugs with fewer side effects compared to conventional treatments. Diabetes mellitus, especially type 2, has become an escalating global health concern. Management of blood glucose levels is crucial for preventing complications associated with the disease. Studies have shown that the ethanolic extract of *S. apetala* fruit possesses hypoglycemic properties, which may be attributed to its ability to enhance insulin sensitivity and reduce oxidative stress [8]. In diabetic animal models, the administration of *S. apetala* extract led to a significant reduction in blood glucose levels, highlighting its potential as a natural remedy for diabetes management. The hypoglycaemic effect of the fruit extract is likely attributed to the presence of saponins and flavonoids [1]. Pain management remains a significant area of research due to the limitations of current analgesic drugs, such as opioids, which can lead to addiction and other side effects. The ethanolic extract of *S. apetala* fruit has shown analgesic effects in animal models, likely through its inhibition of prostaglandin synthesis, a key pathway in inflammation and pain perception [9]. This suggests that the extract could be used as a natural analgesic agent, offering a safer alternative to synthetic painkillers. With the rise of antimicrobial resistance, there is a growing need for alternative therapies to combat bacterial and fungal infections. The ethanolic extract of *S. apetala* fruit exhibits potent antimicrobial properties, effectively inhibiting the growth of multiple pathogenic bacteria and fungi [1]. The antimicrobial effect is likely attributed to bioactive compounds like tannins, alkaloids, and flavonoids, which are known to disrupt microbial cell walls and inhibit the growth of pathogenic microorganisms [10]. This highlights *S. apetala* as a promising source of novel antimicrobial agents with potential to aid in combating the global challenge of antimicrobial resistance.

Medical anthropological studies have demonstrated the significant value of plant-based medicine in treating illnesses and improving lives, much like conventional medicines. Given that plants offer a rich source of bioactive natural compounds, surpassing those found in animals, many individuals have long turned to alternative medicine as a soothing alternative to traditional treatments. Bangladesh, with its diverse array of medicinal plants spanning different families, presents a valuable resource. Consequently, it becomes imperative to conduct rigorous scientific investigations aimed at discovering and developing new, safer medicinal options for addressing various health conditions. As per the

available literature, there have been limited reports regarding the analgesic, hypoglycemic, antidiarrheal, antidepressant, and antimicrobial activities of different fractions derived from this plant. Hence, this research holds immense potential and warrants considerable attention. The primary objectives of this study are to assess the analgesic, hypoglycemic, antidiarrheal, antidepressant, and antimicrobial activities of the soluble fraction within the methanolic extract (MESF) obtained from both the pericarp and seeds of *S. apetala*. Isolation the bioactive compounds present in the n-hexane, ethyl acetate, and chloroform fractions using the GC-MS (Gas Chromatography-Mass Spectrometry) technique.

2. Materials and methods

2.1. *Ethics Statement*

The study protocol received approval from the Institutional Animal Ethics Committee of SUB of State University of Bangladesh (2022-01-12/SUB/IA-ERC/005).

2.2 Sample collection, identification, and authentication procedures

In August 2021, *Sonneratia apetala* fruits were collected from Hatia, Noakhali, Bangladesh [11]. After separating the pericarps and seeds, they were dried, finely powdered, and stored in airtight containers. A voucher specimen (DACB No. 66955) was prepared and preserved following taxonomic identification by a specialist at the Bangladesh National Herbarium in Dhaka.

2.3 Experimental Design, Extraction and processing

The air-dried, powdered pericarp components were soaked in 2.5 litres of methanol at room temperature for 72 hours, with intermittent shaking and stirring, following the established method [1]. Crude extracts were obtained by filtering the mixture through a fresh cotton plug and Whatman No. 1 filter paper, followed by rotary evaporation at low temperatures (below 40°C) under high pressure. The same procedure was applied to the air-dried and powdered seed material (250 g), soaking it in 2.5 litres of methanol at room temperature for 72 hours. Following this, methanol, n-hexane, ethyl acetate, and chloroform extracts were separated by filtration and solvent evaporation at low pressure using a rotary evaporator. Both extracts were precisely weighed and stored under refrigeration. The analgesic, antidiarrheal, antidepressant, and antibacterial properties of the *S. apetala* seed and pericarp methanol extract (ME) were assessed. Bioactive compounds within the n-hexane, ethyl acetate, and chloroform fractions were identified through Gas Chromatography-Mass Spectrometry (GC-MS) analysis. The experimental design for various crude fractions of *S. apetala* fruit is illustrated in Fig. 1.

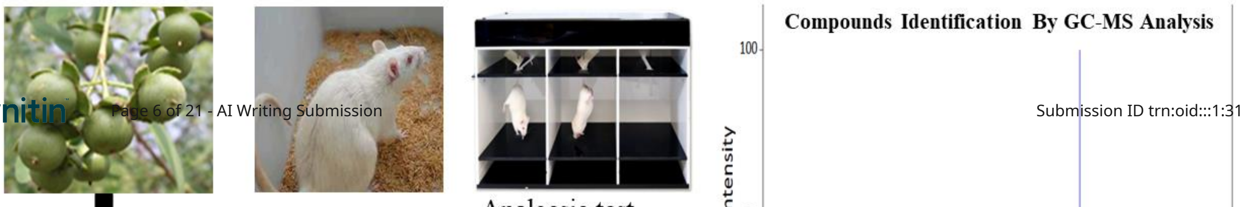

Fig. 1. Experimental Design for different crude fraction of *S. apeta* fruit.

2.4 Animals

Swiss-albino mice, aged between four and five weeks, weighing approximately 23 to 25 grams, and comprising both sexes [12], were utilized in the experiment (see Figure 2). These mice were procured from the International Centre for Diarrhoeal Diseases and Research (ICDDR, B) in Bangladesh. To ensure proper acclimation, each animal underwent a week of adjustment before the commencement of the studies. During the experiments, the mice received a nutritionally complete diet from icddr,b and had unlimited access to tap water. They were kept under standard laboratory conditions with a relative humidity of 55–65%, a controlled temperature of 25.0°C, and a 12-hour light/dark cycle. Prior to each experiment, the mice underwent a 12-hour fasting period to ensure standardised conditions. To minimise discomfort and stress, all procedures followed the guidelines of the Federation of European Laboratory Animal Science Associations (FELASA).

2.5 Model Drugs

The medications used in this study included loperamide (Opsonin Pharma Ltd., Bangladesh), diazepam (Gonoshasthaya Pharmaceuticals Ltd., Bangladesh), glibenclamide (Square Pharmaceuticals Ltd., Bangladesh), and diclofenac sodium (Square Pharmaceuticals Ltd., Bangladesh) [13].

2.6 Central analgesic activity test

The tail immersion method [9], was used to assess central analgesic activity. Mice were divided into four groups of equal size for the experiment: the standard group received diclofenac sodium (5 mg/kg bw), while the control group was administered 1% Tween 80 (10 ml/kg bw). Experimental groups received crude extract doses of 200 mg and 400 mg. In this test, the distal 1-2 cm of each mouse's tail was submerged in water at 55°C, and the latency to tail withdrawal was recorded using a stopwatch. Reaction times were measured at 0, 30, 60, and 90 minutes after oral administration of the test substances, with a maximum latency threshold of 15 seconds. Baseline reaction times were recorded prior to administering any treatments.

2.7 Peripheral analgesic activity test

The writhing test induced by acetic acid is employed to assess the peripheral analgesic activity of the subjects. Writhing refers to body contractions characterized by squirming, and it continues as long as the animals experience discomfort or unpleasant sensations [14]. The mice were evenly assigned to eight groups, each containing four animals. The standard group received diclofenac sodium at a dose of 5 mg/kg bw, while the control group was administered 1% Tween 80 in normal saline at 10 ml/kg bw. Experimental groups were treated with crude extract doses of 200 and 400 mg/kg bw. All mice were intraperitoneally injected with 1% acetic acid, with a 30-minute interval between injections. Group I served as the primary experimental group, and Group III acted as the control group. The positive control group received diclofenac sodium (5 mg/kg), while the control group was given normal saline diluted with 1% Tween-80. The experimental group received test samples prepared with normal saline and 400 mg/kg of Tween 80. To ensure adequate absorption, a 30-minute interval was maintained between the intraperitoneal injection of acetic acid and the oral administration of the test substances. Five minutes after acetic acid injection, the number of writhing movements for each mouse was recorded over the subsequent ten minutes. Additionally, the onset of sleep and total sleep duration were measured for both control and treatment groups.

2.8 Antidiarrheal activity test

The antidiarrheal efficacy of methanolic extracts from *Sonneratia apetala* pericarp and seeds was evaluated using a castor oil-induced diarrhoea model in mice [1]. The study involved four groups of sixteen Swiss albino mice each (n=4). The test groups received the extract at doses of 200 and 400 mg/kg bw, while the control group was given normal saline with 1% Tween 80 at a volume of 10 ml/kg bw. Loperamide, a widely used antidiarrheal agent, was administered to the standard group at a dose of 50 mg/kg bw. Mice were individually housed in cages lined with paper for faecal collection.

To induce diarrhoea, each mouse received 1 mL of castor oil orally, with extracts and loperamide administered one hour prior. Faecal output was observed and recorded over a five-hour period.

2.9 Antidepressant activity test

For the thiopental sodium-induced sleep time test, a modified version of the method [15] was utilised. The study comprised four groups of four mice each. The positive control group was treated with benzodiazepines as the standard, while the test groups received extract doses of 200 and 400 mg/kg bw. The control group was given saline water with 1% Tween 80. Sleep onset and duration were observed for both the control and test groups.

2.10 Hypoglycemic activity test

A glucose tolerance test (GTT) was conducted to assess hypoglycaemic activity. Swiss albino mice of both sexes, aged 4–5 weeks and weighing 23–25 grams, were sourced from the International Centre for Diarrheal Disease Research, Bangladesh (ICDDR, B). The mice were randomly divided into four groups, each with sixteen individuals. Before treatment, the weight of each mouse was accurately measured, and dosages of test samples and control substances were adjusted accordingly. The methanolic extracts of 60 mg and 30 mg were precisely weighed and then diluted in 1 ml of distilled water. A single drop of the suspending agent Tween 80 was added to the mixture. The crude extracts were dissolved in distilled water using a vortex mixer. A feeding needle was employed to administer 0.2 ml of the solution to each mouse in the test groups. To prepare the standard, a 10 mg glibenclamide tablet was dissolved in 3.0 ml of normal saline (0.9% NaCl). Sixty minutes later, all groups received a 10% glucose solution. Blood samples were then collected from the tail vein at 120 and 180 minutes after glucose administration. A glucometer was used to measure the blood sugar levels.

2.11 Antimicrobial Screening

The test samples were dissolved in either chloroform or methanol, depending on their solubility properties. Antimicrobial screening was conducted using the disc diffusion method [16, 17]. Sterile discs were impregnated with diluted samples at a concentration of 400 µg/disc, while ciprofloxacin, at a concentration of 30 µg/disc, served as the standard antibiotic for both antibacterial and antifungal tests.

***2.11.1 Test Organisms, Culture medium and* Composition of Nutrient Agar Medium**

The bacterial strains used in this experiment were obtained as pure cultures from the Institute of Nutrition and Food Science (INFS) at the University of Dhaka. Both Gram-positive and Gram-negative bacteria, as well as fungi, were included in the testing. Gram Positive bacteria such as *Bacillus megaterium*, *Bacillus subtilis*, *Sarcina lutea*, *Staphylococcus aureus;* Gram Negative bacteria such as

Escherichia coli, Vibrio mimicus, Shigella dysenteriae, Pseudomonas aeruginosa, Shigella boydii, Salmonella Paratyphi, S. Typhi, V. parahemolyticus and Fungi such as *Sacharomyces cerevacae, Candida albicans, Aspergillus niger were utilized for testing performance.* Nutrient agar medium was prepared for conducting antibacterial and antifungal sensitivity tests, as well as for cultivating fresh cultures. The medium consisted of the following components per litre: peptone (5.0 g), sodium chloride (5.0 g), beef extract (1.5 g), yeast extract (1.5 g), and agar (15.0 g).

2.11.2 Preparation of the culture medium, subculturing, and the setup of test plates and sample discs

To prepare the required volume of culture medium, calculated quantities of each component were placed in a conical flask, with distilled water added to reach the desired volume. The mixture was heated in a water bath until a clear solution formed, and the pH was adjusted to 7.2–7.6 (at 25 °C) using NaOH or HCl. Portions of 10 mL and 5 mL were then dispensed into screw-cap test tubes to prepare plates and slants, respectively. The tubes were sealed and sterilised by autoclaving at 121 °C under 15 lbs pressure for 20 minutes. The slants were used for cultivating fresh bacterial cultures for sensitivity testing.

Under aseptic conditions in a laminar airflow cabinet, the test organisms were transferred from pure cultures to agar slants using a sterilised transfer loop to establish fresh cultures. The inoculated slants were then incubated at 37 °C for 24 hours to promote optimal growth, after which these fresh cultures were used for sensitivity assays. In a controlled environment, the test organisms were transferred from the subculture to test tubes containing approximately 10 mL of sterilised agar medium using a sterilised transfer loop. The tubes were gently rotated to ensure uniform suspension of the organisms, which was then promptly poured into sterilised Petri dishes. Each dish was rotated clockwise and counterclockwise multiple times to achieve an even distribution of organisms within the medium.

Three types of discs were used in the antibacterial screening. Standard antibiotic discs acted as positive controls to confirm activity against test organisms and to allow comparison with the effects of a known antibacterial agent. Ciprofloxacin discs served as the reference standard. Blank discs were used as negative controls to ensure that neither any residual solvent (remaining after air-drying) nor the filter paper itself displayed antimicrobial activity. Under aseptic conditions, test samples were prepared by dissolving a precise quantity of each compound in a defined volume of solvent to reach the required concentration. The discs were then soaked in these sample solutions and air-dried before use.

2.11.3 Preparation of sample discs with test compounds involved diffusion, incubation, and sterilisation procedures

The antibacterial and antifungal activities of the crude extracts and isolated compounds were evaluated against a range of Gram-positive, Gram-negative bacterial strains, and fungal strains. For crude extract testing, 400 µg of the sample was applied to each disc, and the same quantity was maintained per disc for testing the pure compounds. The sample discs, along with standard antibiotic and control discs, were carefully positioned on designated zones within agar plates pre-inoculated with the test bacterial and fungal strains. The plates were then stored at 4 °C for around 24 hours to allow sufficient diffusion of the substances from the discs into the surrounding agar. Subsequently, the plates were inverted and incubated at 37 °C for an additional 24 hours. To prevent contamination by test organisms, all antimicrobial screenings were performed in a Laminar Flow Hood under strict aseptic conditions. UV light was activated for one hour before beginning work in the hood. Petri dishes and other glassware were sterilised in an autoclave at 121 °C and 15 psi for 20 minutes. Additionally, micropipette tips, cotton, forceps, and blank discs were sterilised prior to use.

2.12 GC–MS analysis for compounds identification

GC-MS analysis was conducted using a GC-MS-QP 2010 Ultra system fitted with an AB Innowax column (30 m × 0.25 mm internal diameter, 0.25 µm film thickness). A 0.5 µL sample was injected, a volume selected likely based on sensitivity, analyte concentration, stability, column capacity, and instrument performance. The oven temperature was initially set at 120 °C for 1 minute, then gradually raised to 270 °C over 25 minutes. Helium was used as the carrier gas with a flow rate of 1.15 mL/min. The split ratio was set to 200, while the injector and mass transfer line temperatures were maintained at 200 °C and 250 °C, respectively. For mass spectrometric analysis, ionisation energy was set at 70 eV. Mass spectra were recorded over a period of 40.75 minutes, with a mass range of 50–650 m/z. The retention indices of the detected compounds were compared with those in the Wiley and NIST Libraries, as well as with values documented in the literature [18].

3. Results and Discussion

3.1 Analgesic activity of the Methanolic Extract of Sonneratia apetala Fruit

Fig. 2 and Table S1 presents the percentage increase in elongation time caused by *S. apetala* pericarp and seed extracts compared to the control. The test samples were then compared to diclofenac sodium for central analgesic activity. The crude methanol extracts of *S. apetala* pericarp and seed demonstrated notable central analgesic activity at doses of 200 mg/kg bw and 400 mg/kg bw, as shown in Fig. 2 and Table S1. The percentage inhibition of elongation at doses of 200 mg/kg and 400 mg/kg by *S. apetala* pericarp and seed methanolic extracts were as follows: 3.69±0.15, 5.30±0.22, and 7.40±0.27; 4.50±0.18, 7.29±0.73, and 10.16±0.59; 6.62±0.19, 5.4±0.38, 7.79±0.23; and 4.06±0.23, 6.04±0.42, and 8.18±0.38 at 30 min, 60 min, and 90 min, respectively. The corresponding values for

the standard and control at 200 mg/kg and 400 mg/kg doses were 5.55±0.19, 10.12±0.33, 17.84±0.41, and 2.28±0.23, 2.27±0.20, and 2.85±0.27 at 30 min, 60 min, and 90 min, respectively. Significant p-values for MESAP (methanolic extract of *S. apetala* pericarp) and MESAS (methanolic extract of *S. apetala* seed) of the pericarp and seed at 200 mg/kg bw doses were found to be 0.002, 0.0002, <0.0001; 0.0022, 0.0014, <0.0001 for 30 min, 60 min, and 90 min, respectively, whereas at 400 mg/kg bw, they were 0.0005, 0.0016, 0.003; 0.0001, 0.0002, 0.0004, respectively. The p-values for the standard (diclofenac sodium) were 0.003, <0.0001, and 0.0001 at 30 min, 60 min, and 90 min, respectively, indicating significant differences. As a result, further investigations can be carried out to develop *S. apetala* fruit extract as a central analgesic medication. This fruit extract has the potential to interfere with the binding of prostanoids to receptors or the metabolic pathway leading to prostaglandin formation. Although it is comparable to the standard medication at a dose of 200 mg/kg bw, the maximum effect was observed at a dose of 400 mg/kg bw.

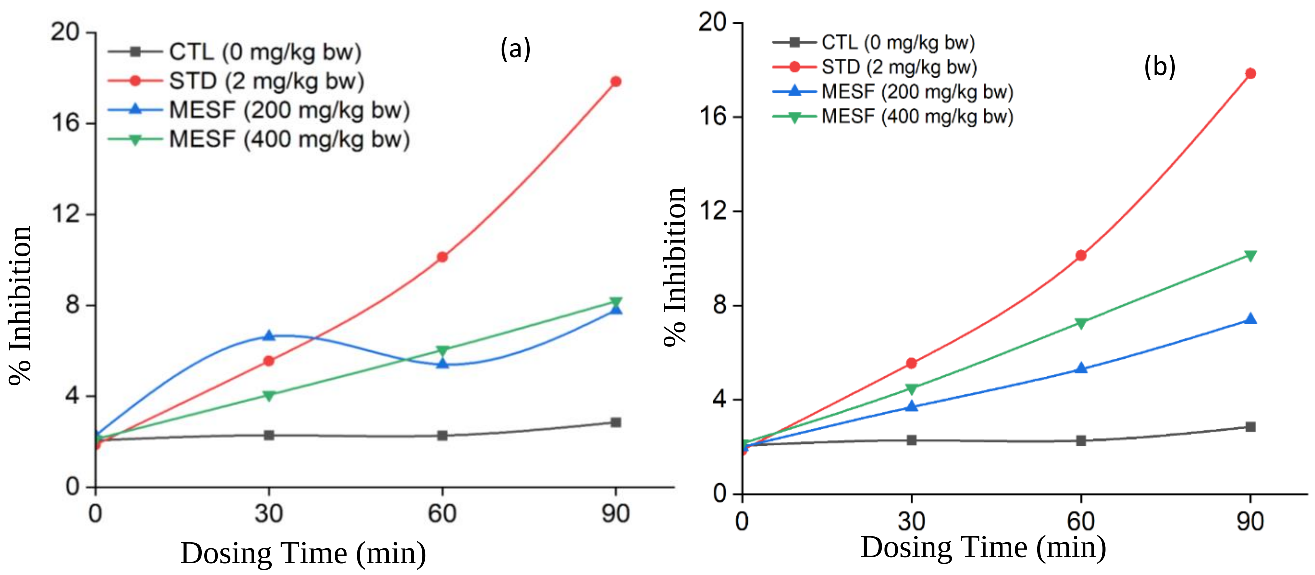

Fig. 2. Evaluation of the central analgesic activity of a crude extract of *Sonneratia apetala* (a) pericarp and (b) seed.

The ME extract may also inhibit neural mediator synthesis [19]. The analgesic properties of *S. apetala* seed extracts were examined using several solvents, including n-hexane, chloroform, ethyl acetate, methanol, and water. Findings revealed that all extracts significantly reduced the frequency of acetic acid-induced abdominal constrictions in mice at a dose of 500 mg/kg body weight compared to diclofenac sodium (25 mg/kg body weight). Notably, the n-hexane extract reduced acetic acid-induced writhing to a lesser extent than the positive control drug, diclofenac sodium (25 mg/kg body weight) [1]. Additionally, during the hot plate test, all fractions exhibited analgesic activity and reduced reaction time. However, only the effects of the diethyl ether fraction remained significant for 180 minutes [1]. In a study examining the analgesic properties of the ethanolic fruit extract using an acetic

acid-induced writhing test [20] observed a dose-dependent analgesic effect. The extract reduced writhing by 46.54% at a dose of 250 mg/kg body weight and by 69.62% at 500 mg/kg body weight.

Table 1. Evaluation of peripheral analgesic activity of crude extract of *S. apetala* pericarp and seed

Group	Dose (mg/kg b.w.)	No. of writhing *MESP*	No. of writhing *MESS*
CTL	0	21±1.82	21±1.82
STD	2	4.5±1.29	4.5±1.29
MESF	200	11.5±1.91	11.5±1.29
MESF	400	8.5±1.73	9±1.82

Each mouse's writhing count was recorded, and the average count for each group was calculated and is listed in Table 1. The percent inhibition of writhing induced by the standard control, *S. apetala* pericarp, and methanolic seed extracts was found to be 4.5±1.29 and 21±1.82; 11.5±1.91 and 8.5±1.73; and 11.5±1.29 and 9±1.82 at 200 mg/kg and 400 mg/kg, respectively. The test samples were then compared to diclofenac sodium for peripheral analgesic activity. The statistical values for the ME and standard were 0.0007, 0.0018, 0.0028, <0.0001, and <0.0001, indicating that the crude methanol extracts of *S. apetala* pericarp and seed exhibited significant peripheral analgesic activity at doses of 200 mg/kg and 400 mg/kg. Therefore, further studies on the plant can be conducted to identify new lead compounds.

3.2 Antidiarrheal activity of the Methanolic Extract of Sonneratia apetala Fruit

As autacoids and prostaglandins play a significant role in causing diarrhea in humans, the castor oil-induced diarrheal model is a valuable experimental approach [21, 22]. The percentage reduction rates, as illustrated in Fig 3 and Table S2, of the ME pericarp and seed at dosages of 200 and 400 mg/kg body weight were 77.78, 90.0, 78.13, and 67.44; and 66.67, 80.0, 78.13, and 72.09 at 200 mg/kg and 400 mg/kg doses at 1 hour, 2 hours, 3 hours, and 4 hours, respectively. Notably, the pericarp of *S. apetala* at a 200 mg/kg dose at 2 hours showed activity almost identical to the standard, while the methanolic extract of the seed at a 400 mg/kg dose at 1 hour exhibited the most potent activity. This indicates statistically significant antidiarrheal effectiveness. The standard and control rates were 100 and 2.25; 95 and 5.0; 97.87 and 8.0; 81.39 and 10.45 for doses of 200 and 400 mg/kg body weight at 1 hour, 2 hours, 3 hours, and 4 hours, respectively. The release of ricinoleic acid from castor oil causes inflammation and irritation of the intestinal mucosa, which subsequently stimulates prostaglandin release, leading to increased motility and secretion [23]. *S. apetala* seeds and fruits have demonstrated significant efficacy as antidiarrheal agents. In a study by [1], young Swiss albino mice were treated with seed extracts from various solvent fractions after inducing diarrhea with castor oil, and a noteworthy inhibition rate of excretion was observed. The inhibition rates for the methanol, chloroform, n-hexane, and ethyl acetate fractions were 68%, 65%, 51%, 50%, and 39%, respectively, at a dose of 500 mg/kg body weight. Additionally, the methanolic fruit extract inhibited defecation by

74.19%, 82.26%, 87.90%, and 94.35% at doses of 62.5 mg/kg, 125 mg/kg, 250 mg/kg, and 500 mg/kg body weight, respectively.

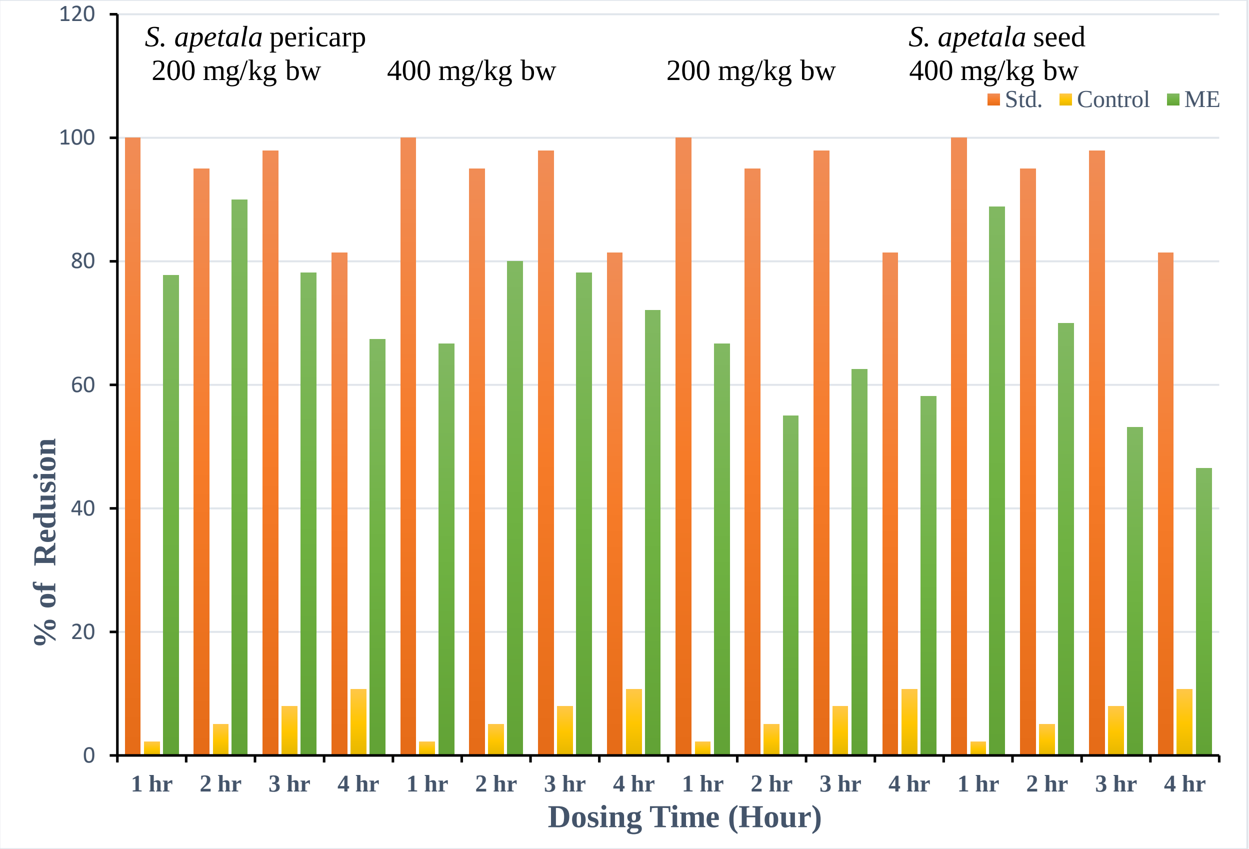

Fig. 3. Antidiarrheal activity of *S. apetala* by the ricinolic acid from castor oil inflamed and irritated method for the pericarp and seed.

3.3 Antidepression Status of the Methanolic Extract of Sonneratia apetala Fruit

The statistical results indicate that methanolic extracts of *S. apetala* pericarp and seed had minor CNS depressive effects at doses of 200 and 400 mg/kg bw, as presented in Fig. 4 and Table S3. The ATOS (Avg. time of onset of sleep) and ATST (Avg. of total sleeping time) standard values were 39.75 min and 79.75 min, respectively, while the control values were also 39.75 min and 79.75 min at doses of 200 and 400 mg/kg body weight, respectively. The ATOS and ATST values for methanolic extracts of *S. apetala* pericarp at doses of 200 and 400 mg/kg body weight were 39.25 min, 68.5 min; 39.25 min, 68.5 min, respectively. Similarly, for the seed extract, the values were 39.25 min, 68.5 min; 39.25 min, 68.5 min, respectively. The P values for the methanolic extracts of *S. apetala* pericarp, seed, and the standard at doses of 200 and 400 mg/kg body weight were found to be 0.04, 0.11, 0.07, 0.19, and 0.4390, respectively (P value is less than 0.5), which is why it was not considered statistically significant. Further investigation is warranted to determine if it has a depressant effect on the nervous

system. It is worth noting that *Sonneratia apetala* bark's methanol extract has demonstrated an average inhibitory effect on membrane stability, indicating its potential for reducing inflammation [22].

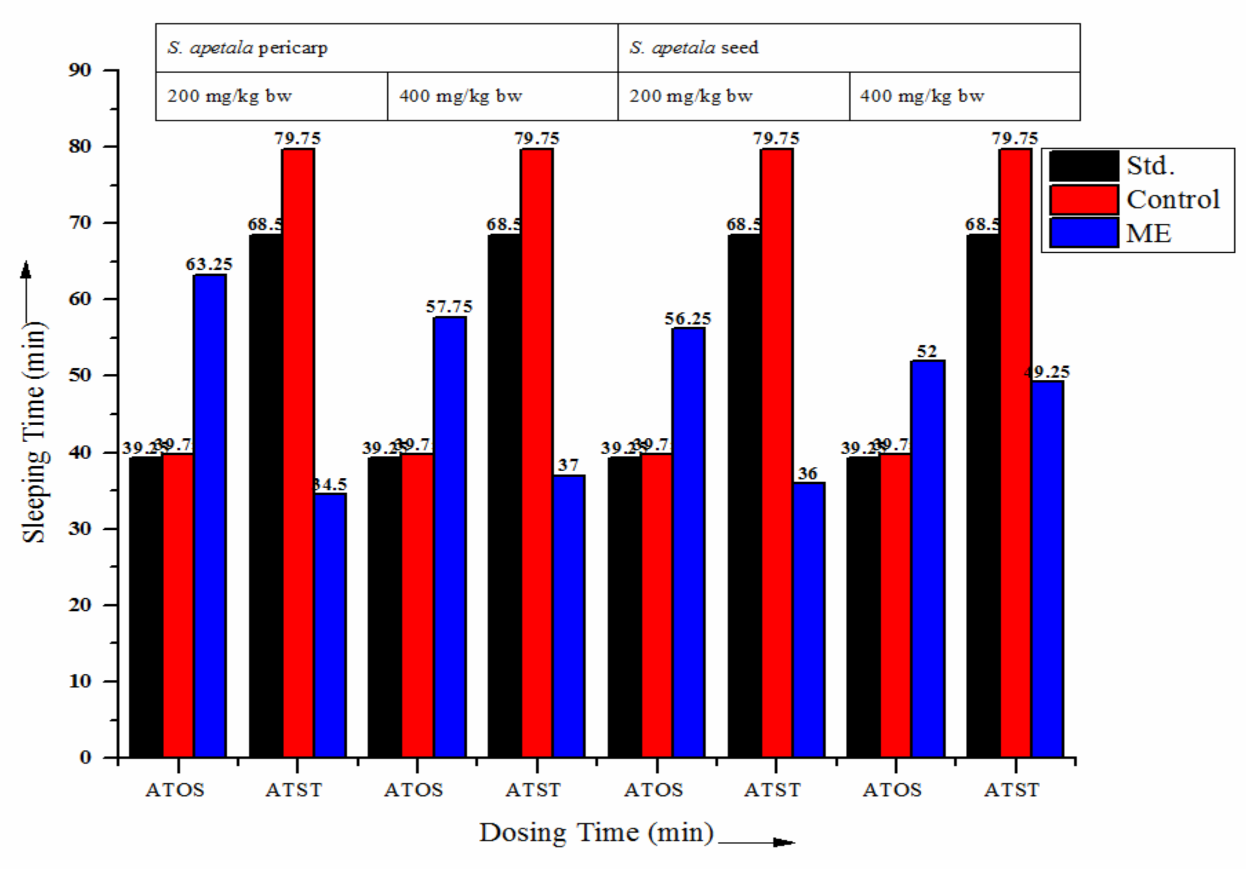

Fig. 4. Antidepressant activity of *S. apetala*by the thiopental sodium-induced sleeping time method for the pericarp and seed.

3.4 Hypoglycemic efficacy of the Methanolic Extract of Sonneratia apetala Fruit

The methanolic extracts of *S. apetala* pericarp and seed demonstrated mild hypoglycemic efficacy, as depicted in Figure 6, at doses of 200 and 400 mg/kg body weight, as shown in Fig. 5 and Table S4. These effects were statistically significant. Plasma glucose levels in the group treated with pericarp extract in streptozotocin-induced type 2 diabetes rats were reduced from 11.43 mmol/L and 16.43 mmol/L at 60 min to 7.35 mmol/L and 10.28 mmol/L after 180 min, respectively. In the group treated with seed extract, glucose levels decreased from 10.7 mmol/L and 11.5 mmol/L at 60 min to 7.25 mmol/L and 7.68 mmol/L after 180 min, respectively. Further research is required in this area to identify potential hypoglycemic lead compounds. The 99% methanolic extract of seeds and pericarp of *S. apetala* exhibited antidiabetic effects (Hossain et al., 2017). After 135 minutes of treatment, serum glucose levels in streptozotocin (STZ)-induced type 2 diabetes rats treated with seed extract decreased from 13.75±2.21 mmol/L at 30 min to 10.3±1.75 mmol/L, while those in the group treated with pericarp extract decreased from 14.36±2.16 to 11.32±1.74 mmol/L (Hossain et al., 2017; Muku

et al., 2016). It is worth noting that the leaf and bark extracts (in methanol) exhibited significantly better antidiabetic potential, with IC_{50} values of 0.286±0.022 and 0.432±0.01 mg/mL, respectively [24].

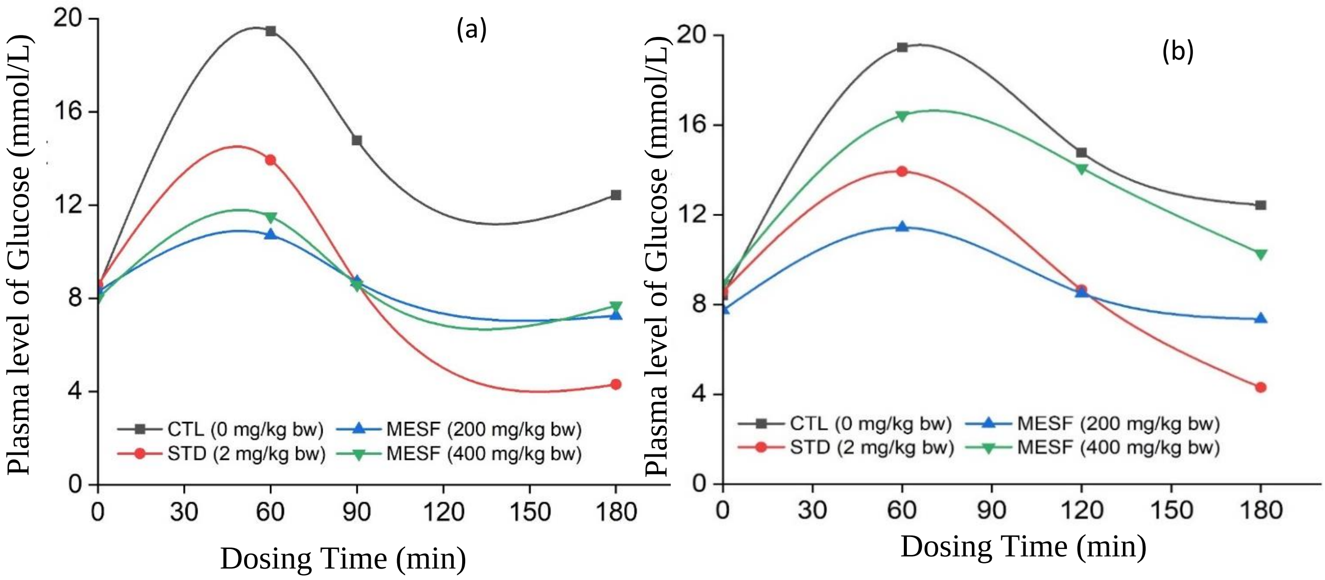

Fig. 5. Plasma level of glucose (mmol/L) of mice at different times for the (a) pericarp and (b) seed of *S. apetala*

3.5 Antimicrobial Status of the Methanolic Extract of **Sonneratia apetala** *Fruit*

The results of the comparison between the antibacterial properties of the methanolic extract and regular amoxicillin are presented in Table 2 and Fig S1. At a concentration of 400 g/disc, the methanolic extract of *S. apetala* seed and pericarp induced zones of inhibition ranging from 9 mm to 15 mm and 10 mm to 13 mm, respectively. These results indicated that the seed methanolic extract had a stronger inhibitory effect compared to the pericarp methanolic extract. In the antifungal assay, both the seed and pericarp of the title plant exhibited strong antifungal activity.

Table 2. Antimicrobial activity of methanolic extracts of the pericarp and seed of *S. apetala*.

Microbial Name	*Std.* Amoxicillin (30 µg/disc)	Methanol extract (400 µg/disc)	
		seed	Pericarp
Gram Positive bacteria			
Bacillus megaterium,	*38*	-	-
Bacillus subtilis,	*40*	-	-
Bacillus sereus	*40*	-	-
Staphylococcus aureus,	*40*	9	*10*
Sarcinalutea	*45*	-	*10*
Gram Negative bacteria			
Escherichia coli,	*40*	-	-
Vibrio mimicus,	*37*	-	-
Shigelladysenteriae,	*45*	-	-
Pseudomonas aeruginosa,	*45*	-	*10*
Shigella boydii,	*35*	-	-

Salmonella Paratyphi,	*40*	*-*	*10*
Salmonella Typhi,	*48*	*10*	*10*
Fungi			
Sacharomycescerevacae,	*38*	*-*	*12*
Candida albicans,	*45*	*10*	*10*
Aspergillus niger	*40*	*15*	*13*

Aspergillus niger displayed the highest inhibitory activity with zones of inhibition up to 15 mm, while *Candida albicans* showed the lowest inhibitory activity with a minimum of 10 mm for both seed and pericarp. Among gram-negative bacteria, *Pseudomonas aeruginosa and Salmonella Paratyphi* were inhibited by the methanolic extract of the pericarp, whereas *Salmonella Typhi* was inhibited by the methanolic extract of both the seed and pericarp up to 10 mm. In the case of gram-positive bacteria, *Sarcinalutea* was inhibited by the methanolic extract of the pericarp, while *Staphylococcus aureus* was inhibited by the methanolic extract of both the seed and pericarp up to 10 mm. The methanolic extract of seeds also demonstrated inhibition against both gram-positive and gram-negative bacteria, including *Salmonella Typhi, Salmonella Paratyphi A, E. coli, Shigelladysenteriae, S. aureus, Proteus species, Pseudomonas species,* and *Shigellaflexneri* [1]. Additionally, the leaf of *S. apetala* exhibited antifungal properties against *Aspergillus flavus and Candida albicans* when extracted with acetone, ethyl acetate, ethanol, methanol, pet ether, and water. However, the acetone extract showed the highest effects [25, 26].

3.5 Bioactive compounds of the n-hexane, ethyl acetate, and chloroform fractions *of S. apetala Fruit*

GC-MS analysis of *S. apetala* fruit revealed seven significant compounds within the n-hexane, ethyl acetate, and chloroform fractions of both the pericarp and seed. These compounds include N-Ethyl-2-methylbenzenesulfonamide, 3,6-Pyridazinedione, 1,2-dihydro-1-(4-nitrophenyl), N-Acetyl-alpha-aminooxybutyric acid (methyl), 2H-Phenanthro[2,1-b]azepin-2-one (1,3,4,5,5a), Benzene (2-ethyl-1,4-diethyl), Benzene (1,2,3-trimethyl), and Undecane. GC-MS analysis identified seven distinct compounds in the n-hexane, ethyl acetate, and chloroform pericarp extracts of *Sonneratia apetala* (see Fig. 6 and Table 3). The structures of these compounds were visualised using ChemDraw software, as illustrated in Fig. 6 (refer to Table 3). The chromatogram for the n-hexane, ethyl acetate, and chloroform seed fractions revealed six prominent peaks, corresponding to the compounds N-Ethyl-2-methylbenzenesulfonamide, 3,6-Pyridazinedione, 1,2-dihydro-1-(4-nitrophenyl), N-Acetyl-alpha-aminooxybutyric acid (methyl), 2H-Phenanthro[2,1-b]azepin-2-one (1,3,4,5,5a), and Undecane, with retention times of 21.38, 16.29, 22.63, 17.48, and 12.3 minutes, respectively. In comparison, the

pericarp extract chromatogram indicated three major peaks, while both ethyl acetate and chloroform fractions in the seed and pericarp extracts exhibited two distinct peaks. These included Benzene (2-ethyl-1,4-diethyl) and Benzene (1,2,3-trimethyl), with retention times of 11.56 and 8.6 minutes, respectively. Table 3 presents the structural details of the predominant compounds identified in the n-hexane extract of *S. apetala* fruit.

Table 3: Tentative Compounds Identified from the Methanol Extract of *S. apetala* by GC-MS Analysis.

Sl No	Name of compound	Fraction	M. weight	M. formula	P. Area	m/z	RT
01	N-Ethyl-2-methylbenzenesulfonamide	nHSF	199.270	$C_9H_{13}NO_2S$	3128	813	21.38
02	3,6-Pyridazinedione, 1,2-dihydro-1-(4-nitrophenyl)	nHSF	233.18	$C_{10}H_7N_3O_4$	5567	387	16.29
03	N-Acetyl-appa-aminooxybutyric acid, methyl	nHSF	386.39	$C_{16}H_{17}F_3N_4O_2S$	2679	656	22.63
04	2H-Phenanthro[2,1-b]azepin-2-one, 1,3,4,5,5a	nHSF	333.5	$C_{21}H_{35}NO_2$	3180	164	17.48
05	Benzene, 2-ethyl-1, 4-diethyl	CSF, CFF	134.22	$C_{10}H_{14}$	3165	119	11.56
06	Benzene, 1,2,3 trimethyl	EASF EAFF	120.19	C_9H_{12}	4733	105	8.6
**07**	Undecane	nHFF	156.31	$C_{11}H_{24}$	2946	57	12.3

N-Ethyl-2-methylbenzenesulfonamide is recognised for its bioactivity, particularly as a potential antimicrobial and antifungal agent [27, 28]. This compound has shown efficacy in disrupting bacterial cell wall synthesis and fungal cell membrane integrity, making it valuable in studies targeting resistant microbial strains. Additionally, its sulfonamide structure allows it to inhibit certain enzymes involved in folate synthesis, a pathway critical for cell division and growth in microbes, thereby exhibiting therapeutic potential in antimicrobial drug development.

3,6-Pyridazinedione, 1,2-dihydro-1-(4-nitrophenyl) exhibits promising bioactivity, particularly as an anti-inflammatory and antioxidant agent [29, 30]. The pyridazinedione core structure contributes to its ability to inhibit reactive oxygen species (ROS) and modulate inflammatory pathways, making it of interest in research related to oxidative stress and inflammation-associated diseases. Studies have shown that derivatives of pyridazinedione, including this compound, can inhibit key enzymes and signaling pathways involved in inflammation, such as COX-2 and NF-kB, which are targets for anti-inflammatory drugs. These properties suggest potential therapeutic applications for this compound in managing chronic inflammatory diseases and oxidative damage.

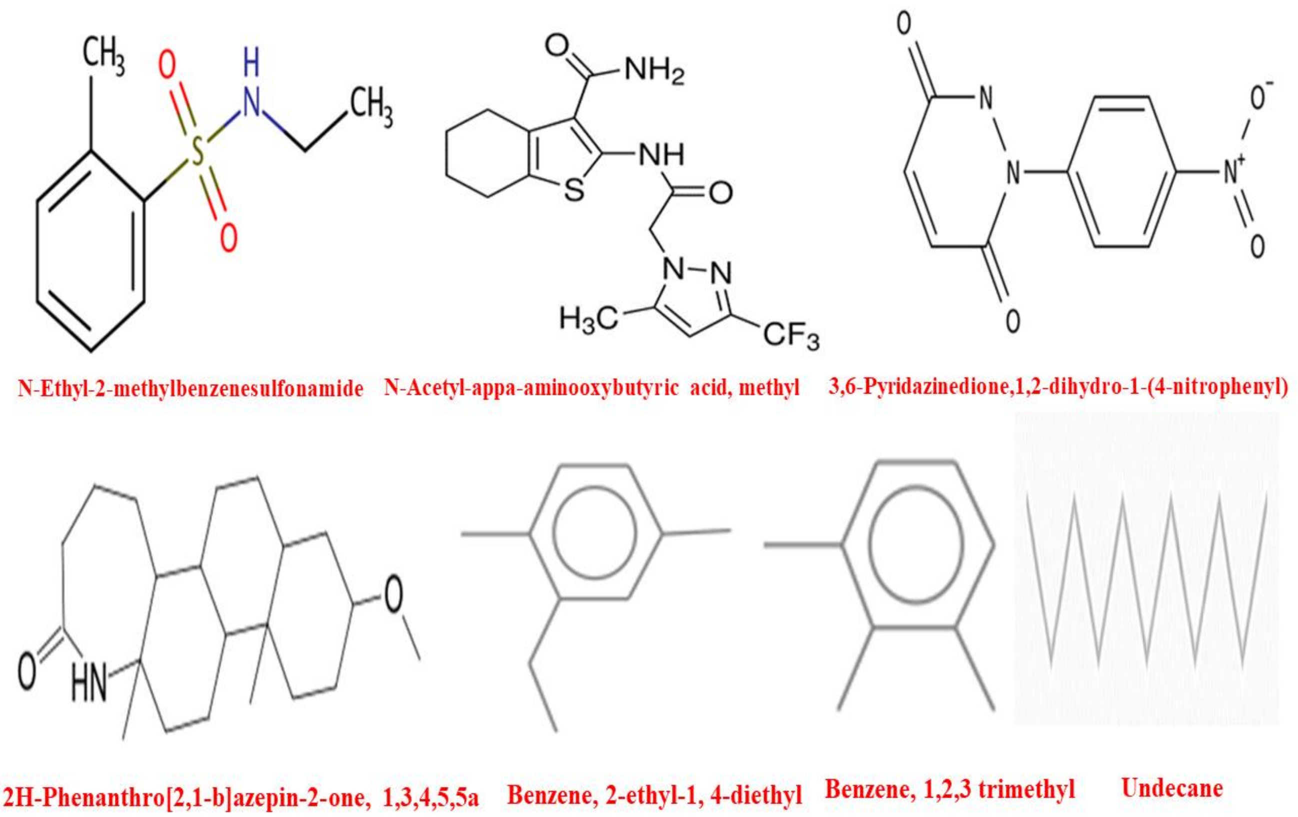

Fig 6: Chemical Structures of the Compounds in the Methanol Extract of the Seed and Pericarp.

N-Acetyl-alpha-aminooxybutyric acid (methyl) is known to exhibit bioactivity primarily in the inhibition of enzyme systems due to its aminooxy functional group. This characteristic makes it effective in inhibiting pyridoxal phosphate (PLP)-dependent enzymes, which play a role in amino acid metabolism. Such inhibition can be valuable in biochemical research, as it allows the study of metabolic pathways by temporarily blocking specific enzyme functions. Additionally, aminooxy derivatives, like N-Acetyl-alpha-aminooxybutyric acid (methyl), have been explored for their antimicrobial and anti-cancer properties [31]. Their ability to modify biological reactions at the cellular level indicates potential therapeutic applications, although further research is needed to fully understand their efficacy and mechanisms of action.

2H-Phenanthro [2,1-b]azepin-2-one (1,3,4,5,5a) has shown notable bioactivity as a potential anticancer and antimicrobial agent [32]. The compound's unique azepine structure allows it to interact with DNA and interfere with the replication process, demonstrating cytotoxic effects on certain cancer cell lines. Additionally, it has been found to inhibit key bacterial enzymes, making it a promising candidate in antimicrobial research. Studies indicate that this compound can initiate apoptosis in cancer cells by disrupting mitochondrial functions, highlighting its therapeutic potential in oncology and infectious disease treatment.

Benzene, 2-ethyl-1,4-diethyl, a type of alkyl-substituted benzene, exhibits limited direct bioactivity but is frequently studied as a structural analogue in hydrocarbon toxicity and environmental health research [33]. Aliphatic-substituted benzene derivatives are commonly assessed for toxicological profiles since alkyl-substituted benzene rings can alter metabolic pathways, leading to potentially harmful metabolites. These compounds are notable for their role in hepatic enzyme induction, oxidative stress, and cellular toxicity, advancing research on environmental pollutants and health impacts. Hemimellitene, or benzene (1,2,3-trimethyl), demonstrates significant bioactivity, with known hepatotoxic and neurotoxic effects due to its conversion into reactive intermediates that induce oxidative stress and DNA damage [34]. This methylated benzene is of interest in toxicology for its influence on liver enzyme activity, promoting free radical formation and cellular damage. Hemimellitene is also under study for respiratory health impacts and as a potential environmental pollutant, significant in occupational and environmental health.

Undecane, a straight-chain alkane, is primarily investigated for its role as a solvent and in environmental studies due to its low reactivity and hydrophobic properties [35, 36]. While undecane itself exhibits limited bioactivity, it can act as a volatile organic compound (VOC) and contribute to atmospheric pollution, with implications for respiratory health upon prolonged exposure. Undecane is also of interest in studies of insect behaviour; it is known to function as a pheromone or signalling compound in some insect species, impacting their aggregation and mating behaviours.

5. Conclusion

Based on the findings of this study, *Sonneratia apetala* fruit demonstrates significant bioactivity with applications in traditional medicine and potential as a source for novel therapeutic agents. The methanolic extracts from both seed and pericarp displayed robust analgesic, antidiarrheal, hypoglycaemic, and antifungal properties, aligning with the fruit's established uses in ethnomedicine. Notably, GC-MS analysis identified bioactive compounds such as N-Ethyl-2-methylbenzenesulfonamide and 3,6-Pyridazinedione, known for their antimicrobial and anticancer effects. In vivo biological research suggests that there is an antidepressant effect, alongside powerful central and peripheral analgesic and antidiarrheal activity. In vivo testing was employed to investigate the effectiveness of the crude methanolic extract of *S. apetala* pericarp as a hypoglycemic agent. Additionally, the extracts exhibited moderate antibacterial activity, showing inhibition against gram-positive and gram-negative bacteria. Additionally, the test for antifungal activity demonstrated that the methanolic extract fraction of seed and pericarp from *S. apetala* exhibited a high level of antifungal activity. Gram-positive bacteria such as *Sarcinalutea* and *Staphylococcus aureus*, as well

as gram-negative bacteria such as *Salmonella Paratyphi*, *Pseudomonas aeruginosa*, and *Salmonella Typhi*, exhibited some inhibitory activities against the methanolic extract of the seed and pericarp. These findings indicate the potential for further investigation of plant materials to identify previously undiscovered efficacy, support their use as traditional medicines, and ideally contribute to the discovery of novel lead chemicals due to their potential bioactivity.

These results indicate that *S. apetala* is a valuable natural source for bioactive compounds with diverse pharmacological effects. Further studies could focus on isolating individual compounds to explore their mechanisms and optimise their therapeutic potential. The findings support *S. apetala* as a promising candidate for developing plant-based therapeutics that could contribute to advancements in medicinal research and provide natural alternatives to synthetic drugs.

Acknowledgments

The authors are grateful to the authority of the Institute of National Analytical Research and Service (INARS), BCSIR, Dhaka, 470 Bangladesh and Ministry of Science and Technology, The people's Republic of Bangladesh for providing analytical, technical, and other logistic support for conducting this research work. The authors extend their appreciation to the Deanship of Scientific Research at King Khalid University for funding this work through a large group research project under grant number (R.G.P.2/20/45).

Data availability: All data are available in the manuscript.

Conflicts of interest: The authors declare no competing interests.

Author Contribution

Fatema Akhter: Methodology, Formal analysis, Investigation, Data curation, Writing-original draft preparation; **Md. Ripaj Uddin:** Conceptualization, Methodology, Formal analysis, Investigation, Data curation, Writing-original draft preparation, Software, Visualization, Writing-Reviewing and editing;**,** **Muhammad Abdullah Al Mansur, Sarker Kamruzzaman and Nahida Akter :** Methodology, Formal analysis; **Mohammad Saydur Rahman:** Methodology and Administration.
