# Supplementary material for: Antidiarrheal, analgesic,antidepressant, antimicrobial and hypoglycemic activities of methanolic extract from Sonneratia apetala fruit, with identification of bioactive compounds in n-hexane, chloroform, and ethyl acetate fractions
Source: PLoS One. 2025 May 5;20(5):e0321280. doi: 10.1371/journal.pone.0321280 (PMC12052150; doi:10.1371/journal.pone.0321280)
Supplement: Attachment — Submitted filename: Reviewer Response.docx [file pone.0321280.s008.docx]

**Reviewer #1:**

Q1: In this manuscript, the authors have investigated the potential medicinal properties of *Sonneratia apetala* fruit, a mangrove species native to Bangladesh. The study focuses on evaluating the analgesic, antidiarrheal, antidepressant, hypoglycemic, and antimicrobial activities of methanolic extracts from both the pericarp and seed of the fruit. The authors also aim to identify the bioactive compounds present in these extracts. This research addresses a relevant topic, as the search for novel therapeutic agents from natural sources is crucial, especially with the rise of antimicrobial resistance. This study employs a comprehensive approach by investigating a wide range of bioactivities, including analgesic, antidiarrheal, antidepressant, hypoglycemic, and antimicrobial effects. This provides a thorough assessment of the fruit's medicinal potential. The use of standard agents like diclofenac sodium, loperamide, diazepam, and glibenclamide as controls strengthens the validity of the bioactivity assays. The study also follows ethical guidelines by obtaining approval from the Institutional Animal Ethics Committee.

Response: Thank you for your nice complement.

Q2: The use of GC-MS analysis enables the identification of specific bioactive compounds, providing insights into the chemical basis for the observed bioactivities. However, the manuscript lacks detailed information on the extraction process, such as the solvent-to-sample ratio, extraction time, and the yield of the extracts. This information is crucial for reproducibility and for assessing the efficiency of the extraction method.

Response: Thank you for your nice observation. In the revised manuscript, we have provided the detailed information on the extraction process (Solvent extraction method), such as the solvent-to-sample ratio (10:1), extraction time (72 hr), and the yield of the extracts (10 gm) at sub section 2.3 in LN 118-140.

Q3: The antidepressant activity assessment relies solely on the thiopental sodium-induced sleep time test, which is not a comprehensive measure of antidepressant effects. Including other behavioral tests, such as the forced swim test or tail suspension test, would provide a more robust evaluation of antidepressant potential.

Response: Thank you for your nice query. We perform Central analgesic activity and Peripheral analgesic activity at subsection 2.6 and 2.7 that is why forced swim test or tail suspension test was not performance. In other work, we will try to cover this test more robust evaluation of antidepressant potential.

Q4: While the antimicrobial activity is assessed against several bacterial and fungal strains, the manuscript doesn't provide the minimum inhibitory concentration (MIC) values, which are essential for determining the potency of the antimicrobial effects.

**Response:** Thank you for your nice suggestion. In the revised manuscript, we have provided the minimum inhibitory concentration (MIC) values of of ciprofloxacin is 0.015 - 2 µg/mL at subsection 2.11 in LN 211.

Q5: The discussion section could be strengthened by providing more in-depth analysis and comparison of the findings with previous studies on *S. apetala* and other medicinal plants.

Response: Thank you for your nice suggestion. In the revised manuscript, we have provided more in-depth analysis and comparison of the findings with previous studies on *S. apetala* and other medicinal plants at section 3 in LN 271-274, 303-306, 316-317, 328-330, 354-356, 371-373, and 395-401.

Q6: The manuscript would benefit from a more detailed discussion of the potential mechanisms of action of the bioactive compounds identified in the GC-MS analysis. Relating the identified compounds to their potential roles in the observed bioactivities would enhance the scientific significance of the findings.

Response: Thank you for your nice suggestion. In the revised manuscript we have added detailed discussion of the potential mechanisms of action of the bioactive compounds identified in the GC-MS analysis at LN 475-509 in sub section 3.5.1.

**Reviewer #2:**

The present manuscript contains 30% similarity and 29% AI generated material.
I recommend authors to clear similarity and AI text and re-submit the article.
This manuscript cannot be proceed and published in present form.

**Response:** In the revised manuscript, the similarity index is less than 20% according to iThenticate software and 0% according to Turnitin software. All AI-generated material has been removed, and the AI report shows 0%.
